# Supplementary material for: Iterative improvement in the automatic modular design of robot swarms
Source: PeerJ Comput Sci. 2020 Dec 7;6:e322. doi: 10.7717/peerj-cs.322 (PMC7924708; doi:10.7717/peerj-cs.322)
Supplement: Supplemental Information 3 [file peerj-cs-06-322-s003.zip › argos3/doc/api/standalone/a00439_source.html]

ARGoS: plugins/robots/foot-bot/control\_interface/ci\_footbot\_distance\_scanner\_sensor.h Source File


- Main Page
- Related Pages
- Namespaces
- Classes
- Files

- File List
- File Members

# plugins/robots/foot-bot/control\_interface/ci\_footbot\_distance\_scanner\_sensor.h

Go to the documentation of this file.

```
00001 
00051 #ifndef CCI_FOOTBOT_DISTANCE_SCANNER_SENSOR_H
00052 #define CCI_FOOTBOT_DISTANCE_SCANNER_SENSOR_H
00053 
00054 /* To avoid dependency problems when including */
00055 namespace argos {
00056    class CCI_FootBotDistanceScannerSensor;
00057 }
00058 
00059 #include <argos3/core/control_interface/ci_sensor.h>
00060 #include <argos3/core/utility/math/angles.h>
00061 #include <vector>
00062 
00063 namespace argos {
00064 
00065    class CCI_FootBotDistanceScannerSensor : public CCI_Sensor {
00066 
00067    public:
00068 
00073       struct SReading {
00074          CRadians Angle;
00075          Real Distance; // in cm from the center of the robot (about 10cm from the border)
00076 
00077          SReading() :
00078             Angle(0.0f),
00079             Distance(0.0f) {}
00080 
00081          SReading(const CRadians& c_angle,
00082                   Real f_distance) :
00083             Angle(c_angle),
00084             Distance(f_distance) {
00085          }
00086       };
00087 
00088       /* These structs are for internal use only */
00089       typedef std::vector<SReading> TReadings;
00090       typedef std::vector<TReadings> TLastReadings;
00091       typedef std::pair<CRadians,Real> TMapPair;
00092       typedef std::map<CRadians,Real> TReadingsMap;
00093 
00097       CCI_FootBotDistanceScannerSensor() :
00098          m_tReadings(4){
00099       }
00100 
00104       virtual ~CCI_FootBotDistanceScannerSensor() {}
00105 
00120       const TReadingsMap& GetReadingsMap();
00121 
00133       const TReadingsMap& GetShortReadingsMap();
00134 
00146       const TReadingsMap& GetLongReadingsMap();
00147 
00148 #ifdef ARGOS_WITH_LUA
00149       virtual void CreateLuaState(lua_State* pt_lua_state);
00150 
00151       virtual void ReadingsToLuaState(lua_State* pt_lua_state);
00152 #endif
00153 
00154    protected:
00155 
00157       TReadings m_tReadings;
00158 
00160       TLastReadings m_tLastReadings;
00161 
00163       TReadingsMap m_tReadingsMap;
00164 
00166       TReadingsMap m_tShortReadingsMap;
00167 
00169       TReadingsMap m_tLongReadingsMap;
00170 
00171    };
00172 
00173 }
00174 
00175 #endif
```

---

Generated on 10 Jul 2018 for ARGoS by 
 1.6.1
